# Short-Term Maternal and Neonatal Outcomes in Preterm (<33 Weeks Gestation) Cesarean Deliveries Under General Anesthesia with Deferred Cord Clamping

**DOI:** 10.3390/children12091151

**Published:** 2025-08-29

**Authors:** Priya Jegatheesan, Gloria Han, Sudha Rani Narasimhan, Matthew Nudelman, Andrea Jelks, Dongli Song

**Affiliations:** 1 Division of Neonatology, Department of Pediatrics, Santa Clara Valley Medical Center, San Jose, CA 95128, USA; sudharani.narasimhan@hhs.sccgov.org (S.R.N.); dongli.song@hhs.sccgov.org (D.S.); 2 Department of Pediatrics, Stanford University School of Medicine, Stanford, CA 94305, USA; 3 Division of Maternal-Fetal Medicine, Department of Obstetrics and Gynecology, Santa Clara Valley Medical Center, San Jose, CA 95128, USA; gloria.han@hhs.sccgov.org (G.H.); andrea.jelks@hhs.sccgov.org (A.J.); 4 Department of Obstetrics and Gynecology, Stanford University School of Medicine, Stanford, CA 94305, USA; 5 Department of Medicine and Clinical Informatics, School of Medicine and Public Health, University of Wisconsin Hospital and Clinics, Madison, WI 53792, USA; matthew.nudelman@hhs.sccgov.org; 6 Valley Health Foundation, San Jose, CA 95128, USA

**Keywords:** deferred cord clamping, delayed cord clamping, duration of cord clamping, umbilical cord clamping, general anesthesia, Cesarean deliveries, very preterm infant outcomes, survival without major morbidities

## Abstract

**Highlights:**

**What are the main findings?**

- A majority (73%) of preterm infants delivered by Cesarean section under general anesthesia received at least 60 s of DCC.
- General anesthesia and DCC were not associated with an increase in delivery room resuscitation or maternal hemorrhage.

**What is the implication of the main finding?**

- DCC can be feasibly implemented in a majority of Cesarean deliveries performed at <33 weeks gestational age under general anesthesia with protocols to shorten DCC time where maternal or fetal safety is threatened.
- DCC during Cesarean deliveries under general anesthesia does not increase maternal hemorrhage or need for neonatal resuscitation and may improve survival without morbidities in very preterm infants.

**Abstract:**

Background: Deferred cord clamping (DCC) is beneficial for preterm infants, but there are concerns about the safety of DCC during Cesarean deliveries (CD) under general anesthesia (GA). We evaluated maternal and neonatal outcomes in preterm CD under GA vs. regional anesthesia (RA) after implementing 180 s of DCC. Methods: This retrospective single-center observational study included CD at <33 weeks gestation, delivered between January 2018 and December 2023. The cord was clamped before 180 s for concerns of maternal bleeding or infant apnea after 30–45 s stimulation. Data was collected from reports from electronic medical records, neonatal intensive care unit database, and manually from the medical records of the patient. Multivariable regression analysis was used to assess the effect of anesthesia type and DCC on outcomes, adjusting for confounders. Results: This study included 170 mothers and 194 infants, and 84.9% of the infants received DCC ≥ 60 s. The GA group had a higher percentage of emergency CD and a lower median duration of DCC (105 s vs. 180 s, *p* ≤ 0.001) compared to RA. In multivariate regression analysis, GA was associated with lower odds (95% CI) of umbilical artery pH < 7 [0.1, (0.0, 0.6)], base deficit ≥ 16 [0.0, (0.0, 0.5)], and higher odds of necrotizing enterocolitis [28.2, (1.4, 560.0)]. GA was not associated with maternal hemorrhage, delivery room (DR) resuscitation, or other major neonatal morbidities or mortality. DCC ≥ 60 s was associated with lower maternal blood loss [Regression coefficient −698, (−1193, −202)], lower odds of transfusion [0.4, (0.1, 1.0)], DR resuscitation [0.4, (0.2, 0.8)], and chronic lung disease [0.4, (0.2, 0.9)], and higher survival without major morbidities [2.8, (1.2, 6.8)]. Conclusions: DCC was performed in a majority of CD under GA by adhering to protocols to shorten DCC in cases where maternal or fetal safety was threatened. GA with DCC was not associated with increased neonatal resuscitation or major neonatal morbidities and was associated with lower maternal hemorrhage and transfusion.

## 1. Introduction

Deferred umbilical cord clamping (DCC), clamping the umbilical cord no sooner than 30–60 s after birth, promotes feto-neonatal transition and placental transfusion, which is critical for short and long-term neonatal outcomes. DCC of at least 30–60 s is recommended by the World Health Organization, the American College of Obstetricians and Gynecologists (ACOG), and the American Academy of Pediatrics for vigorous term and preterm infants who do not require positive pressure ventilation [1,2].

In preterm infants, DCC is associated with improved hemodynamic stability after birth, higher hematocrit, lower risks of blood transfusion, intraventricular hemorrhage (IVH), and necrotizing enterocolitis (NEC), and shorter length of neonatal intensive care unit (NICU) stay [3]. Long-term follow-up studies have demonstrated that DCC is associated with improved motor function at 1–2 years of age [4,5]. Remarkably, DCC is one of the most effective perinatal interventions in reducing mortality in preterm infants. Meta-analyses have shown that DCC reduces mortality by over 30% (odds ratio (OR) 0.68) compared to early cord clamping (ECC) at NICU discharge and at 2 years of age [6,7,8]. A recent network meta-analysis of preterm infants further showed that > 120 s of DCC reduced mortality by 70% (OR 0.31) compared to shorter duration of DCC [9]. We have previously reported that 60–75 s of DCC in very preterm infants, <33 weeks of gestation, reduced the risk of admission hypothermia, intubation, and red blood cell transfusion compared to 30–45 s DCC [10]. More recently, we showed that at least 120 s of DCC improved survival without major morbidities compared to 60 s DCC [11]. Regarding maternal safety, DCC has been shown not to worsen the risk of maternal hemorrhage in vaginal or cesarean delivery (CD) [6,12].

Approximately two-thirds of very preterm infants are born via CD, and 12–50% of these CDs are performed under general anesthesia (GA) [11,13,14]. While various studies have demonstrated the benefits of DCC in preterm infants, most prospective studies of DCC in premature infants have excluded CD under GA, and retrospective studies of DCC have described very few infants delivered under GA. Concerns have included high likelihood for infant resuscitation during emergent delivery and/or infant sedation from GA, as well as increased risk of maternal hemorrhage. A lack of data has led to ambiguity in professional society guidelines and may lead to uncertainty among obstetricians and neonatologists about the feasibility and safety of DCC in this group of high-risk infants.

At our institution, the duration of DCC was increased in a stepwise manner based on the updated literature and internal outcome evaluation. We initiated standardized delivery room (DR) resuscitation, including DCC of 30 s in 2007 [15]. In 2011, the Neonatal Resuscitation Program (NRP) 7th edition guidelines recommended DCC for 30–60 s, prompting us to increase DCC to 60 s. To maximize the benefits of DCC, we progressively increased the goal duration of DCC to 120 s in 2016 and 180 s in 2018. Our DCC protocol did not exclude infants born under GA as this group of high-risk infants may benefit the most from hemodynamic transition stability at birth. To ensure maternal and neonatal safety, we established standardized procedures, contraindications, and indications for early termination of DCC. We implemented an individualized approach to determine the duration of DCC based on open communication among providers who determine if there is a maternal or infant indication to abort DCC at each delivery.

This study aims to evaluate short-term maternal and neonatal outcomes after preterm CD under GA vs. regional anesthesia (RA) after implementing 180 s DCC in our institution.

## 2. Materials and Methods

We conducted a single-center, retrospective cohort study of all CD < 33 weeks gestation performed between January 2018 and December 2023 at Santa Clara Valley Medical Center in San Jose, California, USA. Institutional Review Board (IRB) approval was obtained (#24-007). During the study period, 198 preterm infants were born at <33 weeks gestation by CD and admitted to the NICU. Four cases of lethal fetal anomalies were excluded.

Data on maternal demographics, pregnancy characteristics, delivery data, and maternal and infant outcomes were obtained from standardized delivery reports in the electronic health record and NICU database. Details on anesthesia timing, indication for CD, and indication for GA were confirmed with manual chart review. Anesthesia type was categorized as GA (if GA administration occurred prior to delivery of the infant) or RA, including spinal, epidural, or combined spinal epidural anesthesia. CD urgency was recorded by the delivering obstetrician at the time of surgery; “Emergency/STAT” was defined as need for immediate delivery due to impending fetal or maternal death or injury (e.g., fetal bradycardia). Maternal blood loss was determined quantitatively: amniotic fluid quantity was marked on the suction cannister prior to placental delivery; all surgical drapes, chux, cannisters, and lap pads were weighed at the conclusion of surgery and the weight of dry items and amniotic fluid subtracted. Antenatal steroid (ANS) for accelerating fetal maturation was administered prior to delivery of all infants < 34 weeks; and magnesium sulfate (4 g bolus with or without ongoing infusion 1–2 g/h) for neuroprotection was administered prior to delivery of all infants ≤ 32 0/7 weeks whenever feasible. All CDs were performed by in-house Obstetrics and Gynecology residents and their attending physicians. Anesthesia providers were available in house 24 h a day.

Throughout the study period, our standardized institutional protocol for DCC recommended waiting at least 180 s before cord clamping. In our center, waiting to clamp the cord for at least 60 s after birth was considered DCC and clamping before 60 s was considered ECC. Contraindications to DCC included hydrops and recipient twin in twin-to-twin transfusion syndrome. Additional decisions to abort DCC early were based on discussion between the obstetric and neonatal providers in cases of significant maternal bleeding, cord avulsion, placental disruption, or infant apnea after 30–60 s of tactile stimulation and/or airway suctioning.

Our standardized DR resuscitation and management of preterm infants delivered by CD included the NICU provider being donned in sterile attire to assess and care for the infant immediately after delivery during DCC. Infants were placed on a thermal warming mattress on their mother’s thighs and gentle tactile stimulation, drying, and oral and nasal suctioning with a bulb syringe were provided. A team member verbalized the time in 1 min intervals after birth. Oxytocin infusion was initiated immediately after infant delivery (during DCC). Ring clamps were used as needed to clamp bleeding edges of the hysterotomy during this interval. In multiple gestations, the second-born multiple was delivered before clamping the cord of the first-born multiple whenever possible, thus minimizing the overall duration from uterine incision to closure. If DCC was aborted prior to 180 s, cord milking x4 was recommended, except for extremely preterm infants born at <29 weeks of gestation due to the increased risk of severe IVH [16]. After cord clamping, at the radiant warmer, continuous positive airway pressure (CPAP) was immediately initiated with a T piece and mask with 30% FiO2 for infants < 30 weeks or 21% for infants ≥ 30 weeks gestation. The infant’s temperature was assessed every 5 min, and plastic wrap and other appropriate interventions were utilized to prevent hypothermia during resuscitation [15].

Maternal and neonatal short-term outcomes were analyzed for this study. Outcomes assessed for this study included maternal quantitative blood loss (QBL), postpartum hemorrhage (PPH) defined as blood loss ≥ 1000 mL, and need for blood transfusion. Neonatal DR outcomes included umbilical artery (UA) and umbilical venous (UV) cord blood gas acidosis pH < 7 and base deficit (BD) ≥ 16, intubation, positive pressure ventilation (PPV), CPAP, and cardiopulmonary resuscitation (CPR). Neonatal NICU outcomes included admission temperature, severe IVH, late-onset sepsis, NEC, chronic lung disease (CLD), severe retinopathy of prematurity (ROP), severe neurologic injury including severe IVH and periventricular leukomalacia (PVL), length of stay, NICU death, and survival without major morbidities (late-onset sepsis, CLD, NEC, severe ROP, severe IVH, or PVL).

Neonatal and maternal characteristics were compared between GA and RA groups using univariable generalized estimating equations (GEE), quantile regression, chi-squared tests, and Fisher’s exact tests, as appropriate. Average marginal effects were reported to quantify differences between groups. Neonatal and maternal outcomes were analyzed using multivariable GEE models to evaluate the effect of anesthesia type and DCC ≥ 60 s. Models were adjusted for known confounders including gestational age, antenatal steroid (ANS), emergent CD, and DCC ≥ 60 s. We report effect estimates as odds ratios and regression coefficients. ANS was dropped from several multivariable analyses due to lack of heterogeneity, especially in rare events given the high ANS rate (88–99%) in the study cohort. Umbilical venous pH < 7 and base deficit ≥16 were analyzed using Fisher’s exact test due to sparsity of events.

All infant-based regression models were clustered around pregnancy to account for multiple births. Robust standard errors were applied to all regression models. Linear, logistic, negative binomial, and Poisson models were selected based on the outcome and residual distributions. Descriptive statistics are presented as mean ± SD for normally distributed data, median (IQR) for non-normally distributed data, and n (%) for categorical variables. Statistical analysis was performed using STATA 19.5 (Statacorp, College Station, TX, USA). A *p*-value < 0.05 was considered statistically significant.

## 3. Results

During the study period, 170 mothers underwent CD at <33 weeks gestation, delivering 194 infants. Of these, 30 mothers delivered under GA (33 infants) and 140 received RA (161 infants). The GA and RA groups were similar in maternal age, parity, race/ethnicity, payer, diabetes, severe preeclampsia/eclampsia and chorioamnionitis (Table 1). Emergent CD was significantly more common in the GA group (18/30 (60%) vs. 6/140 (4%), *p* ≤ 0.001). Indications for GA are shown in Table 1. The median (IQR) time from general anesthesia to delivery was 5 (4, 8) minutes. The groups were similar in infant gestational age, birth weight, sex, and ANS. More infants received DCC of at least 60 s (86% vs. 73%, *p* = 0.021) and ≥180 s (53% vs. 21%, *p* = 0.021) in the RA group compared to the GA group.

Table 2 shows the unadjusted analyses comparing maternal and neonatal outcomes in GA and RA groups. The odds of PPH ≥ 1000 mL, DR intubation, CLD, and severe ROP were higher in the GA compared to RA group.

Table 3 shows the adjusted multivariate analysis effects of DCC and GA on neonatal and maternal outcomes after adjusting for gestational age, ANS, and emergency CD. GA was associated with lower odds (95% CI) of UA pH < 7 [0.1, (0.0, 0.6)] and base deficit ≥16 [0.0, (0.0, 0.5)], and higher odds of NEC [28.2, (1.4, 560.0)]. After adjusting for anesthesia type, DCC ≥ 60 s was associated with lower QBL [Regression coefficient −698, 95% CI (−1193, −202)], lower odds of transfusion [0.4, (0.1, 1.0)], DR PPV [0.4, (0.2, 0.8)], CLD [0.4, (0.2, 0.9)], and higher odds of survival without major morbidities [2.8, (1.2, 6.8)].

## 4. Discussion

With a standardized DCC protocol and clear guidelines on when to abort DCC early due to maternal or neonatal safety concerns, more than 70% of very preterm infants born by Cesarean section under GA received ≥60 s DCC in our study. GA was not associated with higher risk of maternal blood loss or adverse outcomes like low APGARs, DR intubation, resuscitation, NICU neonatal morbidities, or mortality. DCC was associated with lower risk of maternal blood loss and transfusions, infant DR PPV, CLD, and higher preterm neonatal survival without major morbidities.

Previous studies have reported that CD under GA is associated with increased adverse maternal and neonatal outcomes compared to RA [13,17,18,19,20,21,22,23,24,25]. The poor outcomes might be attributed to the underlying reasons for performing emergent CD under GA, such as maternal bleeding disorder and/or unassured fetal conditions. In addition, GA itself decreases uterine contraction and may thus increase risk for PPH. Transfer of anesthetic agents across the placenta may cause infant sedation, which poses an additional risk for cardiopulmonary resuscitation. While numerous studies have shown that DCC reduces morbidities and mortality in premature infants, few of the studies included preterm deliveries under GA. Currently, obstetric and neonatal providers face a dilemma in performing DCC in these very high-risk deliveries: DCC facilitates feto-neonatal transition in these most vulnerable infants, yet, in some cases, DCC may worsen PPH and/or interfere with immediate neonatal cardiopulmonary resuscitation. Few recent clinical trials have shown the feasibility of performing neonatal resuscitation during DCC, and more RCTs are underway [26,27,28].

### 4.1. Feasibility of DCC in CD Under GA

Since 2018, our institution has incorporated 180 s of DCC as a part of standardized DR resuscitation of very premature infants with established criteria for contraindications and termination of DCC. In our center, 60–65% of <33 weeks preterm infants are delivered by CD. Notably, our DCC protocol does not exclude CD under GA. During each delivery, the obstetric providers assess for increased maternal bleeding that requires early termination of DCC, and pediatric providers evaluate the infant’s breathing and response to tactile stimulation in the first minute to determine if DCC needs to be terminated to initiate positive pressure ventilation. DCC duration and reasons for early termination of DCC are documented in the delivery notes. As an ongoing quality improvement effort, compliance with the DCC protocol in each very preterm delivery is reviewed at the weekly NICU meeting [29]. This individualized approach has allowed us to achieve a high rate of DCC completion at CD [10,11].

During this study period, almost three-fourths (73%) of infants born under GA received DCC of at least 60 s, with 133 s as the median duration of DCC. This data demonstrates that DCC is feasible in the majority of CD under GA and can be performed in the absence of an indication for earlier cord clamping. Notably, there were more infants in the RA group who received ≥60 and ≥180 s of DCC than in the GA group in this study. This observation is consistent with the literature that GA is associated with a higher likelihood of maternal PPH and the need for immediate neonatal cardiopulmonary resuscitation, which are standardized criteria for early termination of DCC in our center.

### 4.2. Maternal Outcomes

Multiple studies have observed a higher rate of PPH at CD under GA [17,18,19]. However, we could locate no studies reporting on the effect of DCC during preterm CD under GA. In the current study, we notably found that longer DCC duration was associated with lower blood loss and risk of transfusion when controlled for anesthesia type and other important confounders. Taken together with observational and randomized studies of DCC at the time of preterm CD, these studies and ours suggest that DCC itself does not alter maternal blood loss [6,30]. Rather, DCC is more likely to be completed when maternal bleeding is well controlled during CD, regardless of anesthesia type.

### 4.3. Neonatal DR Outcomes

One of the concerns about performing DCC during CD under GA is that the infant’s condition may be compromised while waiting for cord clamping. A recent randomized controlled trial and multiple retrospective studies have shown that APGAR scores are lower and neonatal asphyxia and risk of DR intubation are higher in infants who are delivered by CD under GA, although the cord management strategies were not described in these studies [13,20,21,22,23,24,25]. Another study demonstrated lower 1 min AGPAR and higher FiO2 need in preterm infants delivered under GA with cord clamped at 30 s after birth [31]. In our study, the APGAR scores were similar between the groups. The DR intubation rate was higher in the GA group; however, this was not significant when adjusted for gestational age and emergency CD. This suggests that the higher rate of DR intubation in GA group was likely due to lower gestational age and higher impaired fetal status as the indication for emergency CD.

In the literature, the effect of anesthesia type on cord pH has varied between no difference or lower pH in GA group compared to RA [32,33,34,35]. However, we observed a lower UA cord gas acidosis in GA group after adjusting for confounders. The difference in these DR outcome findings between our study and others may be related to the difference in cord management strategies. In our multivariate analysis, DCC ≥ 60 s was associated with lower risk of needing PPV in the delivery room. Our findings are reassuring that DCC at the time of CD under GA, in cases without maternal and neonatal complications, does not worsen the neonatal status at birth and may improve feto-neonatal transition.

### 4.4. Neonatal NICU and Long Term Outcomes

In previous studies, CD under GA was associated with a significant increase in severe neurological injury and adverse long term neurodevelopmental outcome in infants [13,36,37]. In this study, we found an increase in the risk of NEC but not in other morbidities in the GA group after adjusting for confounders. The incidence of NEC in this study cohort was low (2%). The wide range of 95% CI (1.4, 560.0) for NEC suggests that it is not a precise estimate which may be due to highly variable data and small sample size. This association needs to be evaluated in studies with larger sample size. The rates of CLD and severe ROP were higher in the GA group; however, this was not significant when adjusted for gestational age and emergency CD. This suggests that the higher rates of CLD and severe ROP in GA group were likely due to lower gestational age and impaired fetal status as the indication for emergency CD. In the multivariate analysis, regardless of anesthesia type, DCC was associated with a lower risk of CLD and improved survival without major morbidities. In these cases, DCC may be a surrogate to the overall infant’s state of well-being at the time of birth, or we can speculate that DCC may be playing a beneficial role in minimizing the adverse effects of GA in very high-risk preterm infants.

### 4.5. Strengths and Weakness

To our knowledge, this is the first study that addresses the feasibility and impact of DCC at the time of CD under GA. The strengths of our study include the use of a standardized institutional DCC protocol and high compliance across a 6-year time frame. Specific details pertaining to anesthesia, timing, and indications were able to be verified with manual chart review, ensuring accurate group assignment. Maternal blood loss was assessed quantitatively, rather than by surgeon estimate. Nevertheless, some important limitations must be acknowledged. Our study has a small sample size. Due to retrospective design, our study groups are not matched for important factors known to increase the risk of adverse infant outcomes and PPH. In particular, emergency delivery, non-reassuring fetal status, and maternal thrombocytopenia or bleeding disorder were much more prevalent in the GA group. Because we categorized patients by anesthesia type at the time of infant delivery, some patients in the RA group may have received general anesthesia later in their surgery for maternal indications, possibly affecting their overall hemorrhage risk. UA and UV cord gas values were missing in a third of our study population leading to a smaller sample size and limiting the interpretation of the cord gas data. Lastly, we do not have long-term infant follow-up data.

## 5. Conclusions

DCC of ≥60 s was achievable in a majority of preterm CDs under GA using protocols to shorten DCC in cases where maternal or fetal safety was threatened. In this study, GA with DCC was not associated with increased neonatal resuscitation or major NICU morbidities and had lower odds of maternal hemorrhage and transfusion. This study provides the baseline data for conducting multicenter large-scale studies of DCC in this high-risk delivery to evaluate short- and long-term maternal and neonatal outcomes.

## Figures and Tables

**Table 1:** Demographics.

	General	Regional	Average Marginal Effects (95% CI)	*p*
**Maternal Characteristics**	**N = 30**	**N = 140**		
Maternal age, years *	31 ± 5	32 ± 6	1 (−1, 3)	0.5
Gravity	3 (2, 4)	3 (2, 5)	0 (−1, 1)	1.0
Parity	1 (0, 2)	1 (0, 2)	0 (−2, 2)	1.0
Diabetes or gestational diabetes *	5 (17%)	40 (29%)	13% (−3%, 28%)	0.1
Eclampsia or pre-eclampsia with severe features	12 (40%)	55 (40%)	0% (−19%, 20%)	1.0
Chorioamnionitis *	2 (7%)	15 (11%)	4% (−6%, 14%)	0.4
Emergent cesarean delivery	18 (60%)	6 (4%)	−56% (−74%, −38%)	≤0.001
Classical cesarean section *	15 (50%)	59 (42%)	8% (−12%, 27%)	0.5
General anesthesia indication				
Abnormal placentation	4 (13%)			
NRFHT/need for emergent delivery	18 (60%)			
Maternal thrombocytopenia or bleeding disorder	8 (27%)			
English as preferred language *	19 (63%)	86 (62%)	−1% (−20%, 18%)	0.9
Medicaid or other government insurance *	27 (90%)	120 (87%)	−3% (−15%, 9%)	0.6
Race/ethnicity				0.8
White	5 (17%)	9 (6%)		
Asian or Pacific Islander	2 (7%)	16 (11%)		
Black	2 (7%)	7 (5%)		
Hispanic	21 (70%)	104 (74%)		
Multiracial	0 (0%)	2 (1%)		
Unknown	0 (0%)	2 (1%)		
**Neonatal Characteristics**	**N = 33**	**N = 161**		
Gestational age, weeks	28.4 (25.1, 31.6)	30.1 (27.6, 32.0)	1.7 (−0.6, 4.1)	0.2
<29 weeks gestational age	19 (58%)	61 (38%)	−19% (−40%, 1%)	0.06
Birth weight, g	1201 ± 558	1324 ± 495	119 (−91, 329)	0.3
Birth weight < 1000 g	17 (52%)	54 (34%)	−18% (−37%, 2%)	0.07
Male sex	14 (42%)	88 (55%)	13% (−7%, 32%)	0.2
Multiples	7 (21%)	45 (28%)	7% (−13%, 27%)	0.5
Antenatal steroids	29 (88%)	160 (99%)	11% (−2%, 25%)	0.1
Delayed cord clamping (DCC) duration, s *	105 (45, 153)	180 (101, 180)	75 (39, 111)	<0.001
DCC duration categories				0.02
0–59 s	9 (27%)	22 (14%)		
60–119 s	8 (24%)	22 (14%)		
120–179 s	9 (27%)	32 (20%)		
180+ s	7 (21%)	85 (53%)		
Reason for DCC < 60 s				0.5
Maternal/placental	6 (67%)	18 (82%)		
Neonatal	3 (33%)	3 (14%)		
Both maternal and neonatal	0	1 (5%)		
1 min Apgar	6 (4, 7)	7 (5, 8)	1 (−1, 3)	0.4
5 min Apgar	7 (5, 8)	8 (7, 9)	1 (−1, 3)	0.3

Note: NRFHT—non reassuring fetal heart rate tracing; * Missing data from regional group: Maternal age, Chorioamnionitis, English as preferred language, Classical cesarean section, and Delayed cord clamping duration: n = 1; Medicaid or other government: n = 2; Diabetes or gestational diabetes, Eclampsia or pre-eclampsia with severe features: n = 3.

**Table 2:** Unadjusted Regression Analysis for Outcomes in General vs. Regional Anesthesia.

	General	Regional	Odds Ratio or Regression Coefficient (95% CI)	*p*
**Maternal Outcomes**	**N = 30**	**N = 140**		
Blood loss, mL *	1088 ± 662	881 ± 762	208 (−64, 479)	0.134
Blood loss ≥ 1000 mL	17 (57%)	43 (31%)	2.9 (1.3, 6.6)	0.009
Transfusion	9 (30%)	21 (15%)	2.4 (1.0, 6.0)	0.056
**Neonatal Delivery Room Outcomes**	**N = 33**	**N = 161**		
Delivery room (DR) intubation	5 (15%)	4 (3%)	7.0 (1.8, 26.6)	0.005
DR positive pressure ventilation (PPV)	15 (46%)	50 (31%)	1.8 (0.8, 4.0)	0.1
DR continuous positive airway pressure (CPAP)	29 (88%)	153 (95%)	0.4 (0.1, 1.4)	0.1
DR CPR ^†^	2 (6%)	1 (1%)	na	0.08
DR epinephrine	0 (0%)	0 (0%)	na	na
**Umbilical Artery Blood Gas**	**N = 20**	**N = 96**		
UA pH < 7	2 (10%)	5 (5%)	2.0 (0.4, 11.3)	0.4
UA base deficit ≥ 16	2 (11%)	3 (3%)	3.6 (0.6, 23.6)	0.2
**Umbilical Vein Blood Gas**	**N = 16**	**N = 112**		
UV pH < 7 ^†^	0 (0%)	4 (4%)	na	1.0
UV base deficit ≥ 16 ^†^	0 (0%)	3 (3%)	na	1.0
**Neonatal NICU Outcomes**	**N = 33**	**N = 161**		
Admission temperature, °C	36.9 ± 0.4	36.8 ± 0.3	0.1 (0.0, 0.2)	0.1
Length of stay, days *	54 (33, 95)	47 (27, 78)	12 (−5, 30)	0.2
Death	1 (3%)	5 (3%)	1.0 (0.1, 8.6)	1.0
Severe intraventricular hemorrhage	1 (3%)	4 (3%)	1.2 (0.1, 10.7)	0.9
Late onset sepsis	5 (15%)	9 (6%)	3.0 (0.8, 11.4)	0.1
Necrotizing enterocolitis	2 (6%)	2 (1%)	5.1 (0.7, 36.8)	0.1
Chronic lung disease	13 (39%)	30 (19%)	2.8 (1.2, 6.5)	0.02
Severe retinopathy of prematurity	5 (15%)	4 (3%)	7.0 (1.6, 30.9)	0.01
Severe neurologic injury (Severe IVH, cystic PVL)	1 (3%)	5 (3%)	1.0 (0.1, 8.2)	1.0
Survival without major morbidity/mortality	20 (61%)	116 (72%)	0.6 (0.3, 1.4)	0.2

Note: * Missing data: Blood loss: n = 2 (x1 regional and x1 general); Length of stay: n = 1 (regional); ^†^ Fisher’s exact test was performed due to lack of events.

**Table 3:** Adjusted Multivariable Regression Analysis for Outcomes.

	Adjusted Regression Model *			
	General vs. Regional Anesthesia	*p*	DCC ≥60 s vs. DCC <60 s	*p*
	Odds Ratio or Regression Coefficient (95% CI)		Odds Ratio or Regression Coefficient (95% CI)	
**Maternal Outcomes**				
Blood loss, mL	179 (−194, 552)	0.3	−698 (−1193, −202)	0.006
Blood loss ≥ 1000 mL	2.5 (0.9, 7.0)	0.08	0.5 (0.2, 1.2)	0.1
Transfusion	1.8 (0.5, 6.2)	0.4	0.4 (0.1, 1.0)	0.04
**Neonatal Delivery Room Outcomes**				
Delivery room (DR) intubation	1.0 (0.2, 5.4)	1.0	0.7 (0.1, 4.1)	0.7
DR positive pressure ventilation (PPV)	2.1 (0.7, 6.7)	0.2	0.4 (0.2, 0.8)	0.02
DR continuous positive airway pressure (CPAP) ^†^	0.6 (0.1, 5.2)	0.7	0.8 (0.1, 4.4)	0.8
DR CPR	na	na	na	na
DR epinephrine	na	na	na	na
**Umbilical Artery Blood Gas**				
UA pH < 7 ^†^	0.1 (0.0, 0.6)	0.02	0.6 (0.0, 7.7)	0.7
UA base deficit ≥ 16 ^†^	0.0 (0.0, 0.5)	0.02	2.5 (0.3, 20.6)	0.4
**Umbilical Vein Blood Gas**				
UV base deficit ≥ 16 ^†^	na	na	0.1 (0.0, 1.1)	0.06
UV pH < 7 ^†^	na	na	0.2 (0.0, 1.2)	0.07
**Neonatal NICU Outcomes**				
Admission temperature, °C	0.0 (−0.2, 0.2)	0.7	−0.1 (−0.2, 0.0)	0.2
Length of stay, days	−6 (−14, 2)	0.2	−3 (−10, 4)	0.4
Death ^‡§†^	7.8 (0.8, 80.5)	0.08	na	na
Severe intraventricular hemorrhage ^†^	1.1 (0.2, 6.9)	0.9	0.9 (0.1, 12.0)	1.0
Late onset sepsis ^†^	1.7 (0.3, 10.1)	0.6	0.5 (0.1, 2.2)	0.4
Necrotizing enterocolitis ^‡†^	28.2 (1.4, 560.0)	0.03	na	na
Chronic lung disease ^†^	1.2 (0.4, 3.7)	0.8	0.4 (0.2, 0.9)	0.04
Severe retinopathy of prematurity ^†^	1.7 (0.2, 12.4)	0.6	0.8 (0.1, 5.2)	0.8
Severe neurologic injury (Severe IVH, cystic PVL) ^†^	0.9 (0.2, 4.2)	0.9	1.1 (0.1, 12.0)	0.9
Survival without major morbidity/mortality ^†^	1.5 (0.4, 4.7)	0.5	2.8 (1.2, 6.8)	0.02

Note: * Regression model included anesthesia type, DCC ≥ 60 s, gestational age in weeks, antenatal steroids, and emergency cesarean deliveries (CD). ^†^ Antenatal steroids was not included in the multivariable regression model due to lack of heterogeneity in the events. ^‡^ DCC ≥ 60 s was not included in the multivariable regression model due to a lack of events. ^§^ Emergency CD was not included in the multivariable regression model due to a lack of events.

## Data Availability

De-identified data will be made available by the author upon request.
